# Annular Atrophic Lichen Planus: A Case Report

**DOI:** 10.5826/dpc.1104a123

**Published:** 2021-10-01

**Authors:** Ruzica Jurakic Toncic, Davorin Lončarić, Stefano Caccavale, Joan Garces Reñe, Jaka Radoš

**Affiliations:** 1 University Hospital Centre Zagreb, Department of Dermatology and Venereology, School of Medicine University of Zagreb, Kispaticeva 12, Zagreb, Croatia; 2 Dermatology Unit, Department of Mental and Physical Health and Preventive Medicine, University of Campania Luigi Vanvitelli, Naples, Italy

**Keywords:** annular atrophic lichen planus, dermoscopy, lichen planus, morphea

## Introduction

Annular lichen planus (ALP) is considered one of the rarest clinical variants of lichen planus (LP). It is characterised by a few annular lesions commonly seen on penis and scrotum or in intertriginous areas, and it is usually very resistant to therapy. Annular atrophic lichen planus (AALP) is considered as a distinctive subtype of ALP with histologically extensive elastolysis caused by lymphatic cells [1–2]. Classical dermoscopic criteria for LP diagnosis are missing, diagnosis is therefore challenging, and histological evaluation is needed [1].

## Case Presentation

**Figure 1:**
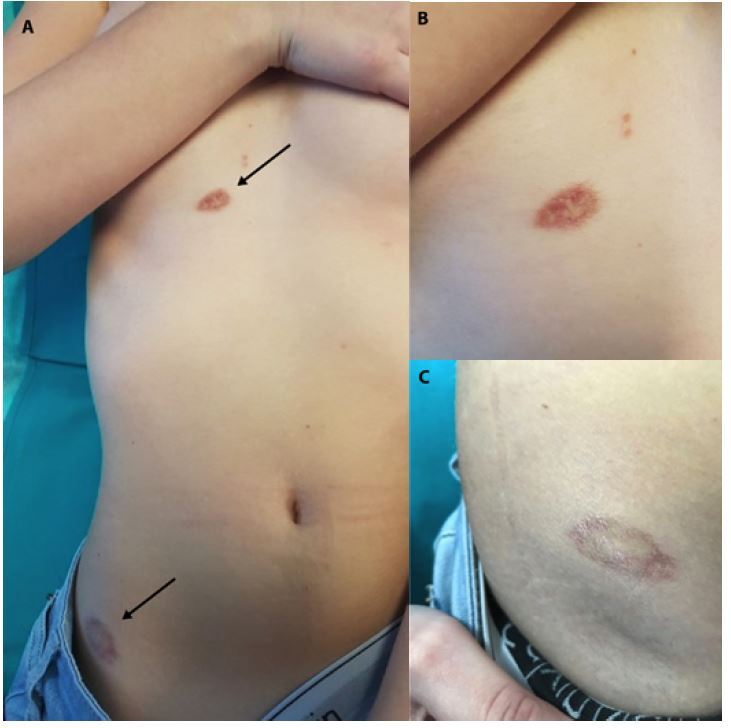
(A) Clinical presentation of both lesions on the trunk, presenting as annular red to livid lesions. (B) Clinical presentation of the first lesion, close up view. (C) Close up view of the second lesion on the trunk.

**Figure 2:**
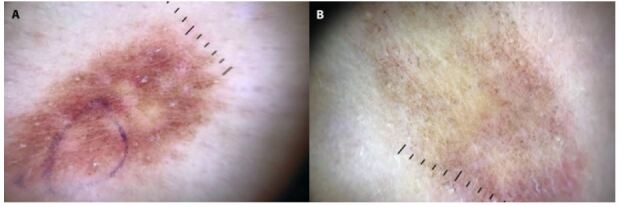
(A) Dermoscopy of the first lesion, presenting with diffuse fine peppering or perifollicular pigmentation. Lesion seems pigmented, with reddish and brownish background. Pigmented pattern corresponds with pigment incontinence. (B) Dermoscopy of the second lesion, which clinically presented as more livid, brownish lesion. Peppering is more pronounced and background appears more gray, yellowish, and pink. Lesion is not sharply delineated at the periphery.

**Figure 3:**
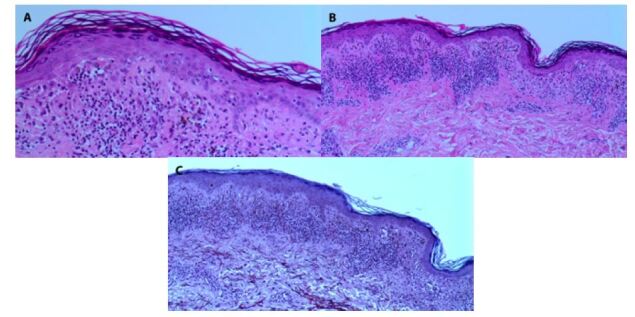
(A) Histology of the lesion (H&E, ×20). (B) Histology of the lesion, details revealing hyperkeratosis, atrophy of epidermis, hydropic degeneration of basal keratinocytes and lichenoid dermal infiltrate with melanophages (H&E, ×40). (C) Histology, orcein stain, showing reduced elastic fibers, finding consistent with AALP.

A 20-year-old female, denying use of any medication or any medical history, sought dermatological examination because of the appearance of 2 asymptomatic lesions under her breast and in her groin fold. Both lesions were annular macules, sharply demarcated, violaceous, or brownish in colour with a shiny surface, measured 20 to 25 mm in long axis (Figure 1). Upon dermoscopic analysis, a pigmented pattern was seen with diffuse fine peppering or perifollicular pigmentation (Figure 2). A more reddish sub-mammillary lesion was biopsied and the histology revealed hyperkeratosis, atrophy of epidermis, hydropic degeneration of basal keratinocytes, and lichenoid dermal infiltrate with melanophages. Elastic fibers were diminished in upper dermis and the final diagnosis of AALP was reached (Figure 3). Laboratory examination showed negative results for both Hepatitis B and C infection. After 1 month of application of high-potency topical steroids, brownish pigmentation and scarring persisted.

## Conclusions

In this variant of LP, characteristic dermoscopic criteria such as Wickham striae and vascular pattern were absent. Of substantial help was the discovery of a pigmented pattern due to pigment incontinence through which differential diagnoses such as morphea or granuloma annulare could be excluded.

## References

[1] Güngör Ş, Topal IO, Göncü EK (2015). Dermoscopic patterns in active and regressive lichen planus and lichen planus variants: a morphological study. Dermatol Pract Concept.

[2] Jose S, Kurien G (2020). Diagnostic dermoscopic features and the correlation between dermoscopic and histopathologic features in lichen planus. International Journal of Research in Dermatology.

